# A Realistic Talking Human Embodied Agent Mobile Phone Intervention to Promote HIV Medication Adherence and Retention in Care in Young HIV-Positive African American Men Who Have Sex With Men: Qualitative Study

**DOI:** 10.2196/10211

**Published:** 2018-07-31

**Authors:** Mark Dworkin, Apurba Chakraborty, Sangyoon Lee, Colleen Monahan, Lisa Hightow-Weidman, Robert Garofalo, Dima Qato, Antonio Jimenez

**Affiliations:** ^1^ Division of Epidemiology and Biostatistics School of Public Health University of Illinois at Chicago Chicago, IL United States; ^2^ Connecticut College New London, CT United States; ^3^ University of Illinois at Chicago Chicago, IL United States; ^4^ University of North Carolina Chapel Hill, NC United States; ^5^ Department of Pediatrics Ann & Robert H Lurie Children’s Hospital of Chicago Northwestern University Chicago, IL United States; ^6^ College of Pharmacy University of Illinois at Chicago Chicago, IL United States

**Keywords:** adherence, mHealth, HIV, African American, men who have sex with men, avatar, embodied agent

## Abstract

**Background:**

Avatars and embodied agents are a promising innovation for human health intervention because they may serve as a relational agent that might augment user engagement in a behavioral change intervention and motivate behavioral change such as antiretroviral adherence and retention in care.

**Objective:**

This study aimed to develop a theory-driven talking avatar-like embodied agent mobile phone intervention guided by the information-motivation-behavioral skills model to promote HIV medication adherence and retention in care in young African American men who have sex with men (MSM).

**Methods:**

We performed 5 iterative focus groups in Chicago with HIV-positive African American MSM aged 18-34 years to inform the ongoing development of a mobile phone app. Participants for the focus groups were recruited from 4 University of Illinois at Chicago Community Outreach Intervention Project sites located in different high HIV incidence areas of the city and the University of Illinois at Chicago HIV clinic using fliers and word of mouth. The focus group data analysis included developing an ongoing list of priorities for app changes and discussion between two of the investigators based on the project timeline, resources, and to what extent they served the app’s objectives.

**Results:**

In this study, 16 men participated, including 3 who participated in two groups. The acceptability for an embodied agent app was universal in all 5 focus groups. The app included the embodied agent response to questions and antiretroviral regimen information, adherence tracking, CD4 count and viral load tracking, motivational spoken messages, and customizability. Concerns that were identified and responded to in the development process included privacy, stigma, avoiding the harsh or commanding tone of voice, avoiding negative motivational statements, and making reminder functions for a variety of health care interactions.

**Conclusions:**

An avatar-like embodied agent mHealth approach was acceptable to young HIV-positive African American MSM. Its relational nature may make it an effective method of informing, motivating, and promoting health behavioral skills. Furthermore, the app’s ease of access, stigma-free environment, and audiovisual format may help overcome some adherence barriers reported in this population.

## Introduction

Young HIV-positive men who have sex with men (MSM) are an important population who may benefit from intervention on the HIV Continuum of Care. MSM account for 82% of new HIV diagnoses among men [1]. The largest subgroup within this population comprises African American MSM. In a national study of HIV-positive MSM reported by the Centers for Disease Control and Prevention, both young MSM (aged 18-34 years) and African American MSM had the lowest viral suppression and retention in care compared with MSM in other age or racial or ethnic groups [2]. Although African Americans represent 13% of the US population, they account for >50% of deaths from HIV or AIDS [3]. African Americans have lower proficient health literacy than Caucasians [4], which is especially important because health literacy is associated with the antiretroviral adherence [5].

An avatar is an animated computerized character designed to look like a person. It may be cartoonish and simplistic or remarkably realistic in its resemblance to an actual person. While avatars are commonly used in computer games, their application to the promotion of human health is new and actively emerging [6-10]. In fact, an avatar is most often used as an embodiment of an online user (as in a computer game); the term embodied agent (or embodied conversational agent) refers to a computer-generated character that provides a feeling of human verbal or nonverbal interaction to the user. Avatars and embodied agents are promising innovative tools for human health intervention because they may serve as a relational agent, a computerized image to which a person may react as if they are in a relationship; this relational aspect might augment user engagement in a behavioral change intervention and thus motivate behavioral change [11]. Avatars and embodied agents offer certain advantages. They may overcome some barriers to care, especially for minority groups, who may be challenged by difficulty traveling to a clinic, have concerns about experiencing stigma in the health care setting, and have other issues such as low literacy and subsequent discomfort clarifying instructions spoken or written above their literacy level.

Avatars and embodied agents can be customized to a user’s preferences, engaging the user in a personal manner. They can engage the user with multiple modalities, such as audio, graphics, text, and interactivity, which may motivate behavior, educate, and encourage repeated use. They also can raise the user’s health literacy by explaining terminology and improving their understanding of the rationale for healthy behavior. Furthermore, encouraging the user with simple engaging speech, hand gestures, facial cues, and other nonverbal behaviors may augment comprehension and impact [12].

Evidence suggests that a mobile avatar health intervention might be effective in African American MSM. Reportedly, African Americans (72%) have high mobile phone ownership [13]. A national survey reported that lesbian, gay, and bisexual persons have higher mobile phone use than heterosexuals [14]. A randomized controlled trial examined a “virtual agent nurse” providing discharge instructions to 764 hospital patients, among whom approximately half were minorities and half had low health literacy [15,16]. The nurse was randomly provided either an African American or a white avatar, and patients exhibited very high satisfaction with the agent. A larger number of patients stated that they preferred talking to the agent compared with a human. Moreover, patients with lower health literacy reported feeling more cared for by the agent than patients with higher health literacy [15,16].

As low health literacy is a factor associated with the antiretroviral therapy (ART) adherence and retention in care [17-22], avatar-based technologies may be a useful vehicle to promote healthy HIV-related behaviors. However, to the best of our knowledge, this is the first study to develop a mobile-delivered, avatar-based intervention addressing retention in HIV care.

We developed a theory-driven, avatar-based embodied agent mobile phone intervention (hereafter referred to as an avatar) to promote HIV medication adherence and retention in care in young African American MSM. This embodied agent was created using an avatar framework from the image of a young African American male volunteer. This mobile phone app was informed by an iterative process of focus groups with young African American MSM that provided feedback on serial versions. The theoretical backdrop for the app design was the information-motivation-behavioral skills model of antiretroviral adherence [23]. Therefore, the app was designed to remedy knowledge gaps (information), improve self-efficacy (motivation), and include functions, such as reminder alerts and calendar functions, which may help improve adherence (behavioral skills). This study aimed to describe the development of this app and lessons learned.

## Methods

### Participants

We conducted 5 focus groups, with 3-4 participants in each group, in Chicago from January to May 2016. Participants for the focus groups were recruited from 4 University of Illinois at Chicago Community Outreach Intervention Project sites located in different high HIV incidence areas of the city and the University of Illinois at Chicago HIV clinic using fliers and word of mouth. In this study, 3 men who participated in the first group returned for the second group, and the other 13 participants were present only one time (*N*=16 participants).

The inclusion criteria for the study were self-reported age of 18-34 years, African American race, MSM, HIV-positive, on ART for at least 3 months by self-report, and owning a mobile phone.

### Procedures

All procedures were approved by the Institutional Review Board of the University of Illinois at Chicago School of Public Health. Before starting each focus group, we obtained informed consent, and participants were provided with a short questionnaire to determine their age, number of ART doses missed in the past 2 weeks, and the main reason for missing doses, if relevant. Of note, focus groups were performed in a confidential setting led by one of the investigators (MD) and an experienced focus group moderator. Focus group locations included Community Outreach Intervention Project sites and the University of Illinois at Chicago's School of Public Health. Participants were encouraged not to share what was discussed within the room or disclose anyone else’s participation, and their names were not used. Focus groups lasted approximately 90 minutes. Feedback from participants was immediately transcribed for review by the research team and discussion with the computer scientist (SL) as developmental drafts emerged. The analysis of the transcribed data was performed by one of the investigators (MD) throughout the project duration. The avatar dialogue was created and edited in response to participants’ recommendations and feedback, and a list of new functions or changes to functions that were explained or demonstrated to participants was created. Then, this list was discussed with the computer scientist (SL), including after each focus group. These two investigators made a decision of which changes to prioritize regarding the feasibility of making each change based on the project timeline, resources needed, and to what extent the change might enhance the efficacy of the app. Examples of app enhancement taken into consideration were would it encourage use, encourage repeat use, and serve the app’s objectives of providing knowledge, motivation, and promoting behavioral skills.

**Figure 1:**
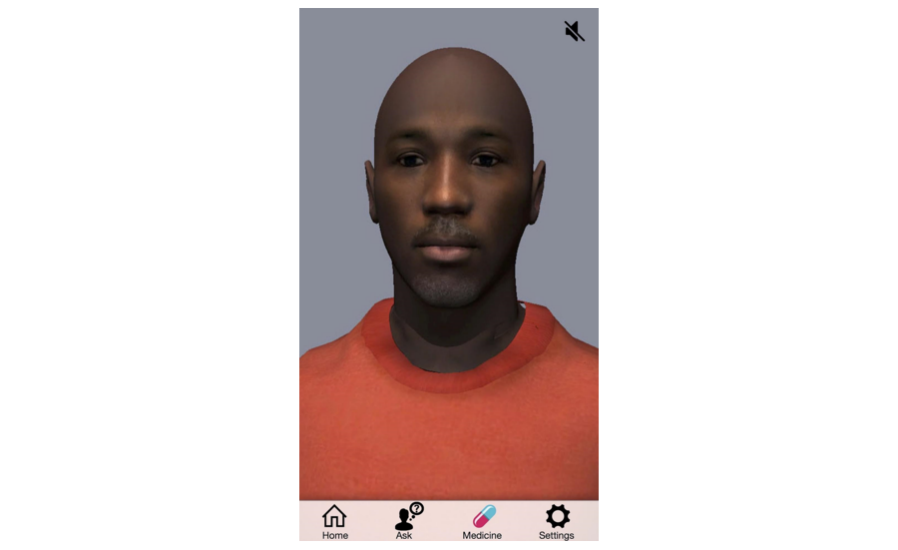
Proposed initial appearance of the avatar shown to focus group participants at the first focus group.

Focus groups began by showing the current form of the app. Then, we asked questions regarding app engagement and each of its proposed functions, which included their opinions about the app symbol, each function in general, and the language used by the avatar. Participants’ likes and dislikes were specifically sought. Then, we asked participants about the proposed functions that might motivate the long-term use and why they had this opinion. Next, we asked them about their perception of the avatar (Figure 1), including its gender, casual or formal appearance, other physical characteristics they sought, and desires for customizability. The discussion progressed to explore what they would like the app to do specifically with respect to helping them take their medication and stay in care. Next, we asked what motivates them to stay in care and what things they would like the avatar to say to motivate them to take their medication without missing doses and stay in care. Furthermore, they were asked whether they wanted the avatar to say anything that acknowledges their dual identity of being both African American and MSM, such as issues of racism, homophobia, beliefs about masculinity, social isolation related to being gay, and HIV status; however, they did not seek such acknowledgment from the app. Finally, they were asked whether they preferred the avatar speaking or did they want to read avatar-provided information. The focus group feedback was categorized as follows to inform the development of the app: acceptability of the intervention (including privacy concerns derived from stigma), avatar customization and content preferences, information, motivation, and behavioral skills that impact HIV care retention. As a dialogue for the avatar was developed (a script), participants were read the dialogue (such as questions that the avatar might say and answer) and then asked about the relevance of the question, comprehension, and reaction to the response. We also asked about other questions they wanted to be addressed. Relational language (ie, language that may promote a social-emotional relationship with the user) was purposively inserted into the dialogue. For example, phrases spoken by participants were inserted into the avatar’s dialogue like “Just keepin’ it real” or referring to something as “nasty.” Similarly, the person chosen to voice the avatar dialogue was an African American man, and his diction was left unaltered when it differed slightly from the written version. Next, new components and content that augmented the avatar’s functionality were developed on the basis of participants’ input and shared with the next focus group for feedback in an iterative way. Initially, participants were shown storyboard images of planned functions and graphics. By the third focus group, the participants were shown the evolving app on a mobile phone, which included both visual and audio components. Although we refer to these gatherings as focus groups, they were not used for identifying themes, and so, no qualitative analyses were performed. Rather, this iterative process was similar to product development by a company that wants to ensure the target population will find the product acceptable. As the purpose of these focus groups was to primarily guide real-time image and function app edits, the data did not require formal qualitative analysis.

### Proposed Mobile Phone App

Entry into the app is password protected. The initially proposed and final version of the mobile phone app includes 4 tabs.

#### Home Screen

Tapping on the first tab delivers the user to the home screen. The avatar greets users on the home screen and provides an audio orientation to the tabs. Later, we added another interaction (inviting the user to hear the motivational messages described below) to the home screen.

#### Let Me Explain

Tapping on the second tab opens the “Let Me Explain” function, which displays the avatar and a scroll bar of questions. Tapping on the question causes the avatar to read and respond. The educational content includes a basic explanation of key concepts relevant to adherence and retention in care such as understanding viral load, CD4 count, routinely obtained blood tests and their rationale as well as what is AIDS, how AIDS differs from HIV, and how often they should see their health care provider. We showed participants draft illustrations that could augment educational content, such as an animation of an x-ray developing an infiltrate, while the avatar explains *Pneumocystis* pneumonia in response to the question, “What can happen if I get AIDS?”

#### Medication Manager

Tapping on the third tab opens the “Medication Manager” function, which includes several tap and click components. Clicking on “Enter Medicine” displays ART and allows users to select their regimen, which the app can display if they click on “My Regimen.” Additional ART information, including generic and commercial names, common side effects, doses per day, and food restrictions, are available by clicking on the image of each medication. Each day the app is opened, the user is prompted on the home screen to record whether they took their ART doses that day. If they respond no, the app asks the reason for missing the dose by providing a list of common reasons to check which applied. If none of the listed responses is applicable, the user can enter a free text response as “Other.” These data results are available to view in a calendar screen available within the “Medication Manager” that shows the image of a pill on the dates with medication taken and an “X” for dates they responded no. “Medication Manager” includes the ability to enter the current and past viral load and CD4 counts to populate a trends curve for each. Later in the iterative process, we added the ability to enter side effects daily.

#### Settings

Tapping on the fourth tab opens “Settings,” where users can create multiple simultaneous personalized reminders for medication and care or lab appointments that can be set as recurring reminders. They may also customize their avatar appearance (such as, hair, glasses, clothes, and background), enter phone numbers of key contacts (health care provider, case manager, and pharmacy) for touch and click calling, and read the “Let Me Explain” and ART information without activating the avatar audio. Within any function, users may pause and silence the avatar with a button located on all screens. When the pause screen is not used, the avatar has a slight head motion and blinking to appear lifelike.

As the app development progressed, participants in the last 3 focus groups were shown the app demonstration on the phone. The avatar’s dialogue included both motivational and empathetic statements (to promote the relationship with the avatar) and phrasing derived from focus group discussions. The avatar dialogue was recorded, not synthetic, and the avatar’s animation included minimal head movement, eye blinking, and mouth movement. By the final focus group, the app offered users with opportunities to respond to questions posed by the avatar, such as whether they wanted to hear the avatar’s (or his friend’s) thoughts on a subject (eg, depression), which would allow the avatar (or his friends in the form of audiorecordings) to provide the additional motivational dialogue that was derived from focus group participant thoughts or recommendations.

## Results

### Participants

The reported ages of 16 participants ranged between 21 and 34 (median 29.5) years. As per the inclusion criteria, all were African American MSM. Of all, 7 had missed at least 3 ART doses, 4 had missed 2 doses, 4 had missed 1 one, and only 1 participant did not miss any ART dose in the past 2 weeks. The main reason for missing a dose was forgetting (n=8), being away from home (n=4), avoiding side effects (n=2), and both being away from home and forgetting (n=2).

### Acceptability

The acceptability for the concept was universal in all 5 focus groups. Participants affirmed that the idea of a talking instructional avatar was a welcome innovation:

He looks so real, and he’s a nice attractive man, and I’m going to ask him a lot of questions about medication! This is genius idea! Thank you! People can go on their phone. People say, I’m afraid to take my meds. This thing can talk back to say, “It’s okay.” Here’s why to take your meds.

Focus group 1

I think it will work. I’m sure it will.

Focus group 2

It’s good that it can explain the side effects. It’s a good idea that there’s a picture of the medicine.

Focus group 3

Participants also provided feedback about the content, suggesting which dialogue should be changed to facilitate better understanding; this informed changes that were tested in a subsequent group that were much better received. For example, although participants accepted the use of the word AIDS in a question such as “Is AIDS the same thing as HIV?”, they reacted negatively to its use as an explanation of the CD4 count by stating, “It’s pretty low if it’s under 200. That’s AIDS if it’s under 200.” A participant asserted that if he had a CD4 count below 200 and was told by the app that he had AIDS as it was stated in this response, it would make him not want to use the app. He stated,

Like I’m gonna keep it real, like if my doctor told me that, I’m not going back to the doctor.

Focus group 4

Another participant said, in response,

I think that in the black community, we are not comfortable with that word, AIDS. Instead, don’t have it say below 200 you got AIDS. Have it say if you less than 200, you got to see the doctor. Don’t say, “That’s AIDS!” Say, “That means your risk of infection is much higher, and it’s so important to take your medication to get healthy.”

Focus group 4

While participants generally welcomed the use of images to complement the dialogue, images portraying sickness or negative consequences of having HIV were less well received. Originally, an illustration of an avatar on a ventilator appeared during the explanation of the complications of AIDS in part to provide a rationale and motivation for healthy behavior. Some participants felt that negative images were upsetting and not motivating. However, illustrations that explained the body and HIV were welcomed such as an image of an x-ray that revealed and explained *Pneumocystis* pneumonia as a complication for AIDS that required taking an antibiotic to prevent it when the CD4 count is low. One participant from focus group 5 stated, “It’s interesting to know what’s going on in the body.”

As a result of the above feedback, the explanation of AIDS was softened to one accepted by the group, “Under 200 means your immune system doesn’t fight infections so good.” Similarly, any image that repelled a participant from wanting to use the app was removed. When no participants agreed that a language or an image was unacceptable, a language that they all could agree was acceptable was offered. A new language was selected when all agreed it was preferable and met the objective of that function (such as clear and educational or motivating). For example, the original wording of the response to the question, “Why do I need my blood drawn?” included the statement, “And some benefits plans that pay for your medication, they need to see your blood test results too.” This was intended to remind them that to keep the benefits of programs, like the AIDS Drug Assistance Program (ADAP), they need to have had their blood drawn, as it is a requirement for every 6-month renewal. However, there were reactions such as, “I just don’t get it,” and another participant said, “That was a distraction, the benefits part.” Subsequently, participants found the following lengthier but more explanatory language acceptable:

“And some benefits plans that pay for your medication, they need to see your blood test results too. Have you heard of ADAP or CHIC? The AIDS Drug Assistance Plan or CHIC Premium assistance? Every 6 months they have to receive your blood results, or they expire and then so does getting medication for free! So to keep certain benefits like ADAP from expiring, you get the blood drawn so the results can get sent in with the renewal application.”

### Stigma and Privacy

Stigma emerged as an important issue in the first 4 focus groups. Specifically, participants were very sensitive to no one knowing their sexual or HIV-related identity through an association with the app. Participants voiced no concerns related to the app by the fifth group, after it had been edited in response to previously voiced issues. Participants sought privacy features that protected anonymity concerning their MSM identity, HIV status, and their health in general. They stated that it was common to have someone looking at or holding their phone. For example, they wanted to minimize the unwanted attention from alerts or reminders to take medicine. Furthermore, they expressed reluctance to ask questions from health care providers or to disclose problems when not asked about them. They suggested a feature that would allow them to instantly change the app screen image to an image that hides their activity to mislead people who are trying to figure out what they are looking at. Notably, images of a game, Facebook, or other culturally appropriate graphics were suggested. A screen hiding function was added by the fourth focus group.

What would be nice, they trying to be nosy and he can change into a game image. Only we know that he’s a puzzle now.

Focus group 1

I wouldn’t want it to pop up saying take your meds.

Focus group 1

The “Did you take your medicine notification” is a problem. “Did you take your medication?” Anybody in their right mind is going be, “What do you mean you take medication?” It lets them know your sick. You could be hiding it from your family.

Focus group 3

In response to the feedback, the avatar tells the user about these privacy features during the orientation while acknowledging an understanding of these privacy concerns in a relational statement: “People are always getting up in my business. How about you?” A relational dialogue, such as this example, may help build trust and credibility as it introduces the avatar as an ally who has some understanding of their world. Specifically, we built a function that allows the user to select among multiple images that would instantly disguise the screen. We also made the reminder alert customizable so the user can decide if they want it to say “take your meds” or a cryptic message only they understand instead.

### Customizability

Nearly all participants found the male avatar acceptable, but 4 stated they would like to be able to choose a female version (specifically a white nurse). They enjoyed the concept of the avatar and desired customization that sometimes reached beyond the budget and timeline for the project. For example, in the fifth focus group, a participant recommended that the avatar have his (the user’s) own face and voice.

Make him functionable like I can brush his hair or take his medicine, drink water, get rest. Have the alarm clock to be aware of his medicine.

Focus group 1

Have everybody create the avatar that they like. That way everybody’s can be distinct. They don’t say, “Hey you got that thing on your phone.”

Focus group 3

Throw a lady in there as a choice.

Focus group 4

Although we considered these recommendations, not all were feasible, given the budget and timeline of the project. Changing the avatar’s clothing, accessorizing him (such as with glasses or a stethoscope), modifying his background (such as office setting), and adding or subtracting hair were built into the app in response to requests.

### Information

Participants were read a dialogue from the “Let Me Explain” component and provided feedback that led to the development of additional content (eg, signs and symptoms of syphilis, taking ART with alcohol, ART side effects, and additional basic HIV and ART adherence information), as well as overall improvements in the dialogue to improve comprehension and relevance. Participants suggested that the avatar dialogue should be combined with imagery to reinforce complicated topics (eg, the meaning of an elevated vs undetectable viral load and affirmed or offered ideas for images).

**Figure 2:**
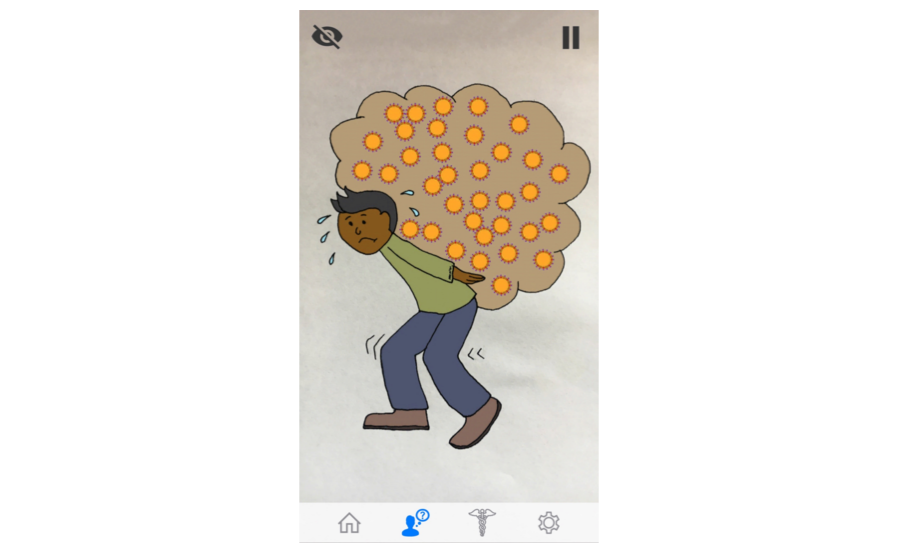
An illustration that appears during the answer to “What is a viral load?” Responding to the question, the avatar explains in a relational way, “You can think of the viral load as the load of virus you’re carrying around. You don’t want to carry around a big load of this virus. Right? So you want the doctor to tell you that your viral load is low.”

The illustrations are a better way of explaining it. Sometimes I still get confused, the CD4 confused with the viral load.

Focus group 2

As a result of this feedback, illustrations were incorporated into several answers (Figure 2).

### Motivation

Participants were enthusiastic about the inclusion of motivational messages that appeared throughout the app, which addressed ART adherence, retention in care, advocating for their needs during appointments, and app function use, including the “Let Me Explain” function’s questions and answers. However, they varied on their preference for positive versus negative encouragement. Some felt that HIV was “like a dog that is barking at you” and can be locked up in a cage by taking medication regularly and that this was a good way to motivate adherence and retention in care. However, others rejected it and favored positive imagery. One participant recommended that taking medication could be thought of like keeping one’s hands on a steering wheel to maintain control, and this language was adopted and found acceptable to the subsequent focus group. Participants also discouraged any intonation in the avatar’s voice that sounded harsh or commanding. They welcomed that the avatar acknowledged their concerns and used “straight talk” to motivate like a friend or relative would. These concerns included advocating for their needs during a medical visit, struggling with adherence, being afraid to take medication, getting help with depression, and asking for re-explanation of health information they did not understand. Furthermore, one participant gave an example of having got clarification about his neuropathy that led to his doctor addressing his problem.

I have neuropathy too. I asked the doctor. He said it come from the disease, not the medicine. If I wouldn’t have asked, I kept going along like it’s ok…I advocated for myself.

Focus group 1

Some people need that push from your parent. My mother said, “boy if you don’t take your medicine I’m not coming to your funeral.”

Focus group 1

“Have avatar tell to take a walk in the park or other motivating language” when depressed. “Some people shuts down.” “See a family member, tell somebody that’s on the same level as me.”

Focus group 1

“When I get any depression, I don’t want to take any medicine.” Regarding avatar dialogue recommending avoiding isolation and activities to try when depressed: “That’ll keep me more motivated. I like that.”

Focus group 2

When someone jumps on my back about something, I get rebellious. Better with a smooth voice, as if they have love for you. My doctor at first irritated me too bad so we almost got in a fist fight. I got up and left.

Focus group 4

Keep your hand on the steering wheel. You got to go straight. You can control your own choice. Its gonna eventually turn. But if you keep your hand on the wheel, you got control.

Focus group 4

In response to the feedback, the avatar specifically motivates the user to advocate for himself. However, it was not clear whether harsh language (referring to one’s funeral) delivered by the avatar (as opposed to a loved trusted person like one’s mother) would be received well so no such language was included. Similarly, the language was edited to be soft, positive, or affirming, and the activities the avatar suggested in response to feelings of depression were those offered by the participants.

In the fifth focus group, participants approved of avatar-spoken motivational statements that would initiate upon entering the app (after its first-time use). The avatar would greet the user with a question such as, “Do you know what I think?” or “Do you want to hear me talk about depression?” If the user clicked yes, the avatar provided a brief statement derived from the focus group discussion such as how social support from a trusted person can help with adherence. In the fifth group, users supported the concept of adding audiorecordings of real people (young HIV-positive African American MSM and health care professionals) giving a brief motivational message. In this study, we recorded 11 people (5 young African American MSM and 6 health care professionals, including 2 doctors, 1 nurse, 1 nurse assistant, 1 pharmacist, and 1 mental health provider) who spoke for 1-2 minutes each. To hear one of these real people recordings, the user would respond to the avatar asking, “Do you want to hear what a friend of mine says?”

Motivational messaging was integrated with many of the “Let Me Explain” responses. For example, when the avatar explains the viral load, language affirming adherence self-efficacy is included. The response to a question about how often to see a health care provider emphasizes the importance of keeping appointments. Similarly, in the final version, an explanation of the benefit of taking HIV medication expresses that “you can handle” the twists and turns of the road of life “by keeping your hands on the wheel,” and an optimistic closing statement follows: “Good luck with your personal goals. Enjoy that full life! You’ll get there.” Participants voiced and offered some of these incorporated motivational phrases or sentences.

### Behavioral Skills

Participants provided suggestions for how the app could address needed behavioral skills to adhere to ART and stay in care. Some participants thought that the ability to track and observe trends in CD4 counts and viral load was “a good idea” but advised that the avatar should “explain how to read the graph.” Participants also approved of reminder functions. However, they encouraged that reminders should be available for other health care events such as blood draws. In response to their feedback, explanatory dialogue concerning the trends and reminder functions for medication taking, appointments, and blood draws were added to the app as choices on a scroll bar, as well as an option of “Other” to accommodate other potential events. A participant recommended a dictation-like function to record concerns to be shared at future health care appointments; this capability was placed on a list for the future work because it was not accomplishable within the 1-year timeframe available for development.

Alarm is a good thing to alert—or to pick up medication too—or to make your appointments, like getting blood work. Have avatar ask about “I’m wondering what’s causing my low platelets.” It could take notes and tell to ask the doctor about this.

Focus Group 1

## Discussion

### Principal Findings

These focus group data demonstrate the value of the iterative development of technology-based health interventions. Enthusiasm for a theory-based mobile phone app that used a talking realistic human avatar-like embodied agent was strong across all five focus groups conducted with young African American MSM. Their feedback helped to design and refine the app for closer alignment with their preferences for the receipt of app-based information, motivation, and skills. By the final focus group, much of the discussion was an affirmation of the more fully developed app and a unanimous request to download the app onto their mobile phones. These findings would help to move the field of adherence research forward. For example, young African American MSM living with HIV liked the idea of a mobile phone avatar-based app approach to adherence and retention in care. While negative messages and images were not welcomed by some young African American MSM, positive messages and images were welcomed by all. In addition, this study revealed that the design of a health app for this population must consider stigma at many levels of interaction (eg, icon, tab appearance, reminders, and password protection).

Many participants described the role HIV- and sexual identity-related stigma plays in their lives and how an app-based intervention should not contribute to this problem. They explained how having an HIV-related app on their phone could place them in situations that could inadvertently result in the disclosure of their HIV status or sexuality. App features, such as an HIV-related icon, reminders, and notifications for the ART use, may draw unwelcome attention. Similarly, if they were to store private information, such as viral load, CD4 count, and medication data, access to the app must be password protected, and time-out features after a period of nonuse were expected. Participants’ input helped to modify the app and strengthen its acceptability, as the icon was made to appear uninteresting, reminders and notifications were designed to be customizable, password entry was required for use, and password entry was required for re-entry after the app automatically closes when the nonuse time reaches 5 minutes. Goldenberg et al conducted focus groups to help develop an app for the HIV prevention in MSM [24]; similar to our study, they found that an app for MSM needed to make them feel safe and was trustworthy. Liu, in a focus group study of patients with chronic diseases in Beijing, reported that worrying about the privacy of personal information was the main reason for not using a health-related app [24,25]. These studies support the prioritization of privacy when designing a mobile phone health app for young African American MSM living with HIV.

A critical part of the development of an avatar or embodied agent intervention is the dialogue. Participants expressed universal enthusiasm for positive motivational statements, whereas the use of negative motivational statements was controversial. Hence, we chose to edit the dialogue to remove negative statements and images that participants could not all agree were acceptable to maximize the acceptability. Accordingly, we replaced these statements with participants’ suggested language and images. While, all participants found educational images of disease acceptable to view (such as a tongue with thrush or an x-ray with pneumonia), images of an ill avatar or statements that allowed one to be directly reminded that they have AIDS were strongly rejected by several participants. Furthermore, negative images can overwhelm users and make then want to turn off the app and not return to it.

An advantage of our approach was that the motivational language could be inserted throughout the app. In the context of the information-motivation-behavioral skills model, motivation is a key component that can drive behavioral change, especially ART adherence [16,26]. Similar to the practice of motivational interviewing, the avatar’s dialogue was intended to move the users away from indecision and toward adherence and retention in care [26]; this kind of language contributes to the relational aspect of the avatar and may allow users to feel that the avatar cares about them.

Among the behavioral skills that mobile phone apps can promote are reminder and notification functions, especially when combined with other interventions and delivered weekly rather than daily [27,28]. Such functions are typically available on mobile phones such as calendar alerts. However, while such capability was already on their phones, participants did not report the common use and welcomed these functions in this app. Although the avatar announces that such functions are available during the initial interaction, it is uncertain whether having the functions in the app will lead to their use or will they be effective. It is possible that encouragement to use reminders in persons who are receptive to them will be most effective if it comes from a health care provider or case manager who may introduce the app to them. Such an interaction might include setting reminders as part of the appointment where the app is used as an intervention. Future research piloting the app will determine the frequency of the use of this function independent from such human assistance. While a notification by itself might be insufficient in producing ART adherence change, this app allows for user customization of the message, and this function was integrated into a broader experience.

Previously, low health literacy has been associated with poor ART adherence and retention in care [17-22]. A talking avatar may help overcome this barrier by providing information in an audiovisual format allowing for an instruction that goes beyond the usual practice in outpatient settings where providers may have limited time for instruction, patients might be reluctant to ask for an explanation, and medical jargon may be used that patients do not understand. Another advantage of a mobile phone avatar app is that information can be replayed to overcome the distraction or emotional impact when a provider says something upsetting and the patient cannot concentrate on the information provided immediately after. In this study, the participants in the focus groups were eager to learn new information and acknowledged that they had learned from the avatar dialogue.

The avatar’s appearance was important to them to ensure he appeared credible. Although they welcomed customizability with casual clothing, they expected him to be dressed in a professional manner as a default. Furthermore, there was a white racial preference by some participants for a female dressed as a nurse. The future app development will offer this option.

### Limitations

The limitations of this study include the generalizability. The focus groups included only 16 participants and were performed in only one city. We focused on young African American MSM because of their relatively high HIV incidence and poorer adherence and retention in care [1,28]. In addition, participants had to own a mobile phone. While the intervention can be modified for delivery on a computer, we do not know whether this broadening of the audience would influence preferences.

### Conclusions

We developed a theory-based, relational realistic talking human avatar-like embodied agent mobile phone intervention to promote HIV medication adherence and retention in care in young HIV-positive African American MSM. We used an iterative approach that helped to ensure that the app development considered the desires and concerns of users. An avatar mHealth approach was acceptable to this population, and its relational nature may make it an effective method of informing, motivating, and promoting health behavioral skills. We propose that this app may be especially helpful if recommended and initially overseen by a case manager or health care provider when a patient is initiating ART or is recognized to be struggling with adherence or retention in care. The next step for this work is a pilot study to determine the preliminary efficacy.

## Abbreviations

ADAP: AIDS Drug Assistance Program
ART: antiretroviral therapy
MSM: men who have sex with men
